# Evidence of Immunomodulatory Food-Protein Derived Peptides in Human Nutritional Interventions: Review on the Outcomes and Potential Limitations

**DOI:** 10.3390/nu15122681

**Published:** 2023-06-08

**Authors:** Fernando Rivero-Pino, Álvaro Villanueva, Sergio Montserrat-de-la-Paz, Susana Sanchez-Fidalgo, Maria C. Millán-Linares

**Affiliations:** 1 Department of Medical Biochemistry, Molecular Biology, and Immunology, School of Medicine, University of Seville, Av. Sanchez Pizjuan s/n, 41009 Seville, Spain; delapaz@us.es (S.M.-d.-l.-P.); mmlinares@us.es (M.C.M.-L.); 2 Department of Food & Health, Instituto de la Grasa (IG-CSIC), Campus Universitario Pablo de Olavide, Ctra. Utrera Km. 1, 41013 Seville, Spain; alvarovillanueva@ig.csic.es; 3 Department of Preventive Medicine and Public Health, School of Medicine, University of Seville, Av. Sanchez Pizjuan s/n, 41009 Seville, Spain; fidalgo@us.es

**Keywords:** antidiabetic, antioxidant, clinical trial, cytokines, inflammation, immune system, protein hydrolysate, validation

## Abstract

The immune system is somehow related to all the metabolic pathways, in a bidirectional way, and the nutritional interventions affecting these pathways might have a relevant impact on the inflammatory status of the individuals. Food-derived peptides have been demonstrated to exert several bioactivities by in vitro or animal studies. Their potential to be used as functional food is promising, considering the simplicity of their production and the high value of the products obtained. However, the number of human studies performed until now to demonstrate effects in vivo is still scarce. Several factors must be taken into consideration to carry out a high-quality human study to demonstrate immunomodulatory-promoting properties of a test item. This review aims to summarize the recent human studies published in which the purpose was to demonstrate bioactivity of protein hydrolysates, highlighting the main results and the limitations that can restrict the relevance of the studies. Results collected are promising, although in some studies, physiological changes could not be observed. When responses were observed, they sometimes did not refer to relevant parameters and the immunomodulatory properties could not be clearly established with the current evidence. Well-designed clinical trials are needed in order to evaluate the role of protein hydrolysates in immunonutrition.

## 1. Introduction

Protein hydrolysates, defined as the pool of peptides of which a protein is composed, are among the most relevant products in the food industry, and may become even more relevant as a key player to improve our food system in the near future, based on the multiple applications they can have, aligned with the ONE health principles and the Sustainable Developmental Goals [1,2]. According to Business Wire, the global protein hydrolysate market size is expected to reach USD 1320.29 million in 2030 and register a revenue compound annual growth rate of 7.30% over the forecast period.

Cleaving proteins to release peptides, aided by food-grade proteases (i.e., enzymatic hydrolysis) or by the action of microorganisms (i.e., fermentation), has been proved to enhance the techno-functional and biological properties of these components [3,4,5]. The properties of peptides are determined by the length, amino acid composition, position of each amino acid in the chain, charge, hydrophobicity, etc. [6]. Improvements in solubility, foaming capacity, water holding capacity, emulsifying properties, and gelling formation among others have been described for protein hydrolysates compared to the native proteins, based on the conformational changes induced during hydrolysis, which favor the exposure of hydrophobic groups [7]. Similarly, numerous food sources have been employed to produce bioactive peptides, including animal and plant proteins, with milk-derived and fish proteins being the most studied ones by far [8]. Regarding other sources, a critical review on immunomodulatory peptides from plant sources; and action mechanisms and recent advances have been recently published [9]. Among the bioactivities more widely studied and described in literature, the antioxidant and antihypertensive properties of peptides can be highlighted [5,10,11], as well as anti-inflammatory or antidiabetic [12,13], though most of the reports published until now are in vitro assays and consequently, the scope and relevance of these studies are limited. This in vitro demonstration of bioactivity is not enough to determine the health-promoting activities of peptides but might serve as a screening of a huge number of candidates. In terms of in vitro studies, usually spectrophotometric methods are employed, which have led to the publications of a huge number of reports claiming the bioactivity of food-derived peptides, potentially exerting effects in different metabolic pathways. However, these results cannot be extrapolated to their behavior in organisms, and the claim in literature of bioactive peptides being health-promoting with these studies is misleading and overused without sufficient evidence, including how different metabolic pathways could be correlated and affected or how the inflammation status of the organism could be modified based on indirect changes in the physiology. In fact, one of the main limitations of in vitro studies is the lack of information concerning stability of peptides during gastrointestinal digestion, absorption in the lumen, and their reaching to the target organ and actual bioactivity exerted. Usually, in situ cell-based and/or ex vivo analysis may narrow the gap between the in vitro assays and the in vivo studies, but still these cannot be considered as highly relevant evidence [14]. Several authors have aimed to define the molecular features making a peptide bioactive towards specific targets [15,16], but considering the high amount of sites where peptides can bind the target enzymes on which they exert the activity, a single sequence could potentially exert diverse bioactivities [17]. This is relevant in terms of the real effects that peptides would have, as in vitro assays only claim that peptides exert usually specific bioactivities, though they might have an impact on different target sites. Nonetheless, as aforementioned, these health-promoting properties these peptides might exert are hindered by their bioavailability, as it has been widely reported that these peptides are usually not reaching the targets due to the sequence’s susceptibility to be cleaved during the absorption, distribution, and metabolism process [18]. On the other hand, animal studies can only be used for investigating potential mechanisms and modes of action, and even when the results add the weight of evidence of the immunomodulatory properties that peptides might exert, it has to be taken into consideration that the metabolism of animals (e.g., mice, rats, rabbits) is not fully comparable to that of humans [19,20].

Inflammation is the immune system’s reaction to a harmful stimulus, triggering a cascade of events promoting healing of the affected tissue [15]. Signaling pathways are activated as part of the inflammatory response, leading to the release of inflammatory mediators, including interleukin-1 beta (IL-1β), IL-6, and tumor necrosis factor alpha (TNF-α). The immune system is related to several physiological processes and targets that are related to the development of diseases. This specific information will be detailed in the following section. On top of that, nutrition has been proved to exert several effects on humans by regulating metabolic pathways at different levels. The potential to modulate the activity of the immune system by interventions with specific nutrients is termed immunonutrition [21]. Foods can alter immune functions manifesting as either innate immunity (phagocytic activity, NK cell activity) or acquired immunity (T cell response, antibody production) [22]. The immune system has been described to be positively affected by the consumption of vitamin C, iron, selenium, and omega-3 fatty acids, evidenced by lower incidences and milder courses of infection [23]. However, protein hydrolysates as bioactive components with immunomodulatory potential are currently a field at an early stage of research in which methodologies have to be improved and results obtained are not conclusive.

The regulatory requirements of bioactive peptides (protein hydrolysates) from food proteins change in different regions. In terms of the market of bioactive protein hydrolysates, a summary of commercial products currently available on the market with health benefits based on bioactive peptides/protein hydrolysates can be found elsewhere [8]. However, health claims must be granted by assessing well-defined human studies with a significant population size and with physiological changes clearly proved, in order to determine the real effectiveness of molecules in the population. At European Level, for instance, a bonito protein hydrolysate was considered a negative opinion for maintenance of normal blood pressure [24]. In the EU Register on nutrition and health claims, no protein hydrolysates were found as being authorized when this manuscript was written. Nonetheless, in Japan, for instance, products containing peptides from fish (bonito and sardine) obtained the “Foods for Specified Health Use” (FOSHU) approval with demonstrated angiotensin-converting enzyme inhibitory activity, and consequently, can be sold as blood pressure lowering dietary supplements [8].

**Figure 1:**
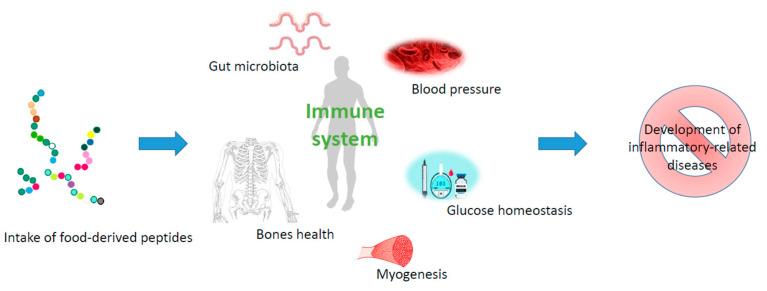
Schematic representation on how nutritional interventions with peptides have a role in immunonutrition.

Assessing whether a protein hydrolysate can have an important effect on the immune system is challenging, considering the endpoints to be measured and how this system is correlated with several tissues and organs (Figure 1). Taking this into account, in the following section, human studies investigating potential health-promoting effects of protein hydrolysates recently reported are discussed, and how these results can be correlated with changes in the immune system of the individuals is investigated.

## 2. Requirements to Demonstrate Health Benefits

The demonstration of health benefits on humans because of the ingestion of food components must rely on human studies, rather than in vitro or animal studies. In any case, it is on the weight of evidence that the health claims can be stated and will depend on experts’ judgement. This review should not be considered as such but rather the aim is to explore the potential methodological problems that studies can present. A full investigation on which limitations apply is not in its scope, as full reports of the studies are not published, and we considerate it unfair to judge the quality of the experimental design and the reporting of the results.

Overall, one of the main problems that clinical trials have is that they are expensive, and it is a lot harder to control every variable, so the results may not be reliable nor applicable to a wider population. In fact, the difficulty of statistical analysis is considered to be a major challenge, especially in cases of extremely heterogeneous patient groups [25], particularly concerning the immunological status of the individuals, which is highly variable, related to genetics and environmental factors [26]. Prior to the design, a power calculation of the sample size (of a suitable group) has to be conducted in order to provide a sufficient strength of evidence, as it affects the hypothesis and the study design [27].

Regarding the type of study, ideally, the nutritional intervention should be performed by conducting randomized controlled trials (i.e., at a low risk of bias). Uncontrolled, cross-sectional, or exploratory studies, or improperly randomized studies, cannot be used to state that the intake of specific food components have a biologically relevant influence on individuals. In addition, the conditions of use might be realistic; thus, the route of administration has to be orally, considering amounts of the test item that are reasonable in a normal diet, and it has to be taken into consideration that the upper level (if any) for vitamins and minerals is not surpassed. Considering that nutritional interventions should be safe for human beings, adverse effects have to be recorded, based on the conditions of use. Focusing exclusively on the benefits while ignoring the potential risks associated with the intake of peptides can be misleading when formulating and following dietary recommendations [28]. Ideally, a dose–response relationship should be established to demonstrate that the test item is responsible for the physiological changes in the subjects, as well as the specificity and magnitude of the effect for the duration of the treatment [29].

An important factor is also the consistency of the effect across studies (reliable results obtained from studies by independent research groups and/or in different locations strengthen the evidence). For this, an evaluation of comparable studies, identified by carrying out a systematic review methodology as default, is needed. It is important in this regard to also carry out an evaluation of studies with completely contrary results, which would not support the potential health claim of a specific test item [30].

When preparing the human study as such, the most important thing is to define all the parameters involved (dose, duration, target population, factors to be evaluated, etc.). Concerning the parameters to be assessed, it is important to, prior to the analyses, define and justify the election of primary and secondary outcome(s), and then to perform a correction for the testing of multiple outcomes. One relevant factor to be taken into consideration is to always include baseline data in the statistics. The baseline data provide substantiation on the external validity of the study, as it allows the comparison among results from different studies. In terms of the intervention as such, where relevant baseline factors are well balanced, it is more probable that any changes in outcomes between the intervention and control groups are a consequence of the treatment (one component of internal validity) [31]. In the election of parameters, it has to be considered that not all results that can be measured in vivo in humans by generally accepted methods reflect a direct benefit on human physiology. The differences between the control group and the study group should ideally be only the test item whose health benefits want to be demonstrated, as the cause-consequence can be linked to the test item as such, while if the intervention is in a background where the rest of the food ingested is not sufficiently controlled, the relevance of the results is lower and the definition of the health benefit might not be clear enough.

Regarding the duration of the nutritional intervention, some parameters (e.g., blood lipids or blood pressure) tend to stabilize after about 4 weeks in response to fixed nutritional interventions. However, the time needed to reach such maintenance may depend on the study traits and the nature of the intervention. Consequently, evidence concerning the stability of the impact of continuous consumption of the food/constituent over longer periods of time (e.g., 8 weeks) is required according to guidelines in order to properly substantiate the effects. However, these changes on their own cannot be used as a factor to determine that the nutritional intervention has an impact on the immune system, even when there is a close relationship between several parameters and the inflammation status.

Sometimes, it is possible that the statistical analyses performed in order to explore health benefits are not appropriate, as they might not account for the repeated measures design or the multiplicity of secondary outcomes. In some cases, studies only report statistical analyses for completers while not reporting some data or not analyzing data that are needed in order to support the fact that the benefits can be linked to the consumption of the food. On top of that, a multi-center design of the study is not always considered in data analysis [32].

To sum up, a careful preparation of the human study intended to be performed, an adequate realization of the assay, and a clear and critical statistical analysis of the results obtained are needed in order to consider a human study as being relevant enough to draw conclusions.

## 3. In Vivo Studies: Description of Results

### 3.1. Background

The source of protein hydrolysate that is most studied historically is milk-derived proteins, especially whey. In this regard, there are several recent reports aiming to demonstrate several physiological changes by the ingestion of peptides. In this section, a summary of recent studies (from 2018 to 2023) is described, strictly indicating the results as reported from the authors concerning biological parameters analyzed related to some diseases (e.g., hypertension or diabetes). Then, the relationship between the risk factors or diseases described with the immune system has been also mentioned, aiming to link the results and to establish the, if any, role of protein hydrolysates in immunonutrition.

### 3.2. Effect on Inflammatory Markers

The effect of peptides on different anti and/or pro inflammatory markers has not been widely studied in humans. These compounds can be found in several cells and tissues, acting as signals to other cells and tissues in the body. This complexity makes it difficult to draw conclusions on that based on single experiments. IL1-α, IL1-β, IL6, and TNF-α are pro-inflammatory cytokines, whereas IL-10, IL-13, and IL-4 are considered anti-inflammatory. Furthermore, the gene expression of these is not always correlated with their expression as proteins [33]. In addition, the immune status of the subjects, whether healthy or not, has an impact on the baseline data of the individuals, hindering the comparison within a group in order to establish mechanisms of actions.

Rein et al. [34] reported that the acute intake of 20 g of rice protein hydrolysate by healthy subjects (n = 10, mean age of 36 years old) implied a temporary mild modification of cytokine levels in plasma. In this regard, a small suppression of pro-inflammatory cytokines (e.g., TNF-α, interferon gamma-induced protein 10 (IP-10), and NOx) was reported, at the same time that IL-6 increased temporarily (timepoints 2–12 h). However, according to the authors, these markers returned to the baseline after 24 h whereas others were not affected significantly, including IL-10, high-sensitivity C-reactive protein (hs-CRP), IL-8, and monocyte chemoattractant protein-1 (MCP-1). Nonetheless, the experimental data could not confirm the bioavailability of the peptides which were considered bioactive by prediction analyses, but results suggest that response is most likely through intestinal signaling.

Laatikainen et al. [35] evaluated whether a milk protein hydrolysate could ameliorate gastrointestinal symptoms in subjects (n = 41, mean age of 44 years old) suffering from gastrointestinal disorders, when ingested for ten days. These authors also collected blood and urine samples in order to evaluate markers of inflammation, intestinal permeability, and immune activation (including TNF-α, IL-6, fatty acid-binding protein 2 (FABP2), and 1-methylhistamine), though no significant changes were reported following the nutritional intervention. In a similar study with cod protein hydrolysates, Dale et al. [36] evaluated patients (n = 13, mean age of 43 years old) suffering from irritable bowel syndrome (IBS) and whether the intake of cod peptides could have a positive effect on symptom severity, gut integrity markers, pro-inflammatory cytokines in serum, and fecal fermentation, as the inflammatory response in non-healthy subjects was expected to be different. The intervention lasted for six weeks, during which 2.5 g of the test item/day was ingested, and no significant variations in concentrations of IL-8, lipopolysaccharide binding protein (LBP), iFABP, or zonulin were reported. Regarding effects, one subject indicated an increase of IBS symptoms related to diarrhea and/or pain correlated with the ingestion of the supplement.

In the same line, Jensen et al. [37] showed that, in a randomized, double-blind study with adults suffering from metabolic syndrome (n = 15, mean age of 53 years old), the supplementation with 4 g of cod peptides during eight weeks did not induce any statistically significant differences in the concentration of the inflammatory markers evaluated (i.e., TNF-α and IL 1β, IL 6).

The diversity of the results found hinders the possibility at the moment to draw global conclusions, considering the limitations that the studies herewith reported show. Nonetheless, the not-so promising results hereby reported, considering that most of the studies reported no significant changes, could potentially be re-evaluated with enhanced methodologies or different conditions of the assay.

### 3.3. Effect of Peptides on Bones, Muscles, and Joints

The relationship of the immune system with bone is reciprocal, meaning that bone cells also influence immune cells. In this regard, bone cells are responsible for creating the so-called “endosteal niche”, as these cells are implied in the mobilization of hematopoietic stem cells [38]. Similarly, in terms of muscles, it has been described how the variations in the phases of myogenesis during muscle regeneration after having suffered from an injury concur with changes in the phenotype and activation state of leukocytes that invade the damaged, regenerating tissue. In fact, TNF, interferon-γ (IFNγ), (IL-10), and insulin-like growth factor 1 (IGF1) play an important role in managing the inflammation and the myogenic response to muscle damage that is required to achieve muscle regeneration [39].

Koning et al. [40] reported how collagen peptides, under the circumstances assessed, improved bone mineral density and bone markers in postmenopausal women, through a randomized, placebo-controlled, double-blinded study. Over one year, postmenopausal women (n = 131, mean age of 64 years old) with a primary, age-related reduction in bone mineral density, ingested 5 g daily of the test item (collagen peptides) or a placebo, as the control. The primary endpoint was the change in bone mineral density of the femoral neck and the spine. On top of this, plasma levels of bone markers (i.e., amino-terminal propeptide of type I collagen and C-telopeptide of type I collagen) were also analyzed. According to the authors, the subjects who ingested the test item showed an increased bone mineral density of the spine and of the femoral neck in comparison to the control group. Concerning the markers, the propeptide increased significantly in the peptide-ingesting group, whereas the C-telopeptide was found to be higher significantly in the control group. This is similar to when chicken collage type II was ingested (2.5/day) over 8 weeks, and the target population, consisting of adults with joint discomfort, but no co-morbidities (n = 47, mean age of 66 years old), were found to have a significant reduction in joint-related discomforts [41].

Brown et al. [42] aimed to assess whether a whey protein hydrolysate (WPH) supplementation could be useful for exercise-induced muscle damage and recovery in females (n = 20). The stimulus was a repeated-sprint exercise, and then the subjects ingested two doses of 70 mL of the test item (whey protein hydrolysate) or a control consisting of isoenergetic carbohydrate, over 4 days post-exercise. The endpoints measured were muscle soreness, limb girth, flexibility, muscle function, and creatine kinase, which were evaluated before, immediately after, and 24, 48, and 72 h post-exercise. According to the authors, time effects were observed for all variables except limb girth. It was observed that flexibility improved beyond baseline measures following WPH by 72 h and that the reactive strength index was higher throughout recovery in the test item group compared with the control. In addition to that, reductions in creatine kinase were higher following the intake of whey hydrolysate compared with the carbohydrates at 48 h post-exercise-induced muscle damage.

Concerning fish proteins, Nygard et al. [43] aimed to demonstrate the benefits of cod peptides in older adults (n = 86, mean age of 73 years old) who ingested 3 g for a year. Parameters such as short physical performance battery, grip strength, gait speed, and dietary intake were considered, but at the end of the study, authors reported no significant differences observed after the test item ingestion.

Funglsang-Nielsen et al., [44] evaluated the effects of 12 weeks of whey protein supplements on markers of bone turnover in adults with abdominal obesity. For this purpose, a population of adults with abdominal obesity (n = 64, mean age of 66 years old) ingested 60 g of peptides daily. The main endpoints evaluated were plasma markers of bone turnover, as well as u-calcium and u-carbamide excretion, bone mineral density, and insulin resistance. However, no major changes were observed in the parameters assessed, except for a slight increase of parathyroid hormone levels. In the same line, recently Kerr et al. [45] evaluated whether a Vicia faba hydrolysate, which showed beneficial muscle recovery and anti-inflammatory properties by in vitro and animal studies, was effective in humans. For this purpose, a randomized, double-blind, placebo-controlled trial with healthy males (n = 30, mean age of 38 years old) was carried out, in which the subjects ingested 2.4 g of the test item/day for two weeks. According to the results, strength recovery was increased, and muscle fatigue decreased compared to the placebo over the 72-h period that post-resistance exercise was observed. In addition to that, other acute markers increased such as IL-6, IL-15, or Erythropoietin (EPO).

In studies related to inflammation and recovery of bones and/or muscles, a relevant parameter to be taken into account is the age of the individual, as ageing has a strong impact on the physical status of humans, deteriorating the organism and increasing the susceptibility of the individuals to suffer from inflammation. More parameters related to inflammation could be useful in order to unravel the mechanism of action by which the peptides exert the effects observed in the reported studies.

### 3.4. Effect on Gut Microbiota

The relationship between gut microbiota and the immune system is clear, although it is not fully described and characterized nowadays. Briefly, maintaining intestinal homeostasis and reducing inflammation depend heavily on the dynamic interactions between the gut microbiota and the host’s innate and adaptive immune systems. Proteins and complex carbohydrates are metabolized by gut microbiota, implying a communication between gut epithelium and immune cells. Gut epithelial cells create a mucosal barrier as a protective measure to separate the bacteria from host immune cells and lessen intestinal permeability. A compromised epithelial barrier and a greater susceptibility to infections can result from an abundance of potentially harmful Gram-negative bacteria and the metabolic changes they cause in the gut microbiota and mucosal immune system [46].

Recently, the alteration of gut microbiota by bioactive peptides was reviewed by Guo et al. [47] and Yeo [48], among other authors, but most of the studies refer to in vitro assays or animal studies and are thus not in the scope of this review.

In terms of human studies, Moreno-Perez et al. [49] carried out a randomized pilot study in cross-country runners (n = 12, mean age of 35 years old). The subjects followed, over ten weeks, a diet supplemented with a whey isolate and beef hydrolysate, whereas the control was maltodextrin. The microbiota was evaluated by collecting fecal samples and the results showed an increase of the Bacteroidetes phylum, whereas a decrease of Roseburia, Blautia, and Bifidobacterium longum, usually related to good health, was reported, thus implying a negative effect according to these parameters.

Furthermore, the body composition, gut microbiota, and serum metabolomics of old women (n = 60, mean age of 61 years old) with an energy-restricted diet were evaluated following an intervention consisting of 20 g of whey peptides over 8 weeks. The main outcomes were a reduction in body weight and the upregulation of the tricarboxylic acid cycle pathway, but no significant changes were reported in the microbiota [50].

Further studies aiming to evaluate how the ingestion of food-derived peptides from different sources affect the gut microbiota are needed in order to fully understand whether the impact can be considered biologically relevant, and the consequences derived from that. The differences found among studies are most likely due to the background diet, especially fiber, which has a relevant effect on microbiota, as well as the protein composition ingested and the high interindividual variation among subjects.

### 3.5. Effect on Glucose Homeostasis and Blood Lipids

Regarding diabetes, which is also one of the most prevalent diseases worldwide, it has similarly been demonstrated how inflammation plays a key role in the advancement of metabolic abnormalities, and bidirectionally, that metabolic factors modulate immune cell functions. An impairment of the metabolic status of subjects has an impact on the immune cell infiltration and inflammation while also implying an increase in reactive oxygen species. The consequence is the development of insulin resistance [51], while at the same time, it has an impact on gut microbiota dysbiosis, which is related to an alteration of the functions of innate and adaptive intestinal immunity [52]. The effects of cholesterol-lowering dietary compounds on the immune system have been widely reviewed, as well as the impact of cholesterol on molecular and cellular events and subsequent biological responses of immune cells [53]: for instance, initiating NLRP3 inflammasome-mediated cascades [54] or inducing apoptosis by ROS production. Peptides appear as a relevant component in managing diabetes-related risk factors.

The safety and efficacy, for instance, of a beverage containing lupine (*Lupinus angustifolius* L.) peptides on the immune, oxidative, and lipid status in healthy subjects was evaluated in an intervention study (open-label, n = 33, mean age of 30 years old). The daily dose was 1 g, ingested over 4 weeks, and implied changes in several biochemical parameters in fasting peripheral blood and urine samples. This increased the anti-/pro-inflammatory response of peripheral blood mononuclear cells and improved the cellular anti-oxidant capacity. The test item also reduced the low-density lipoprotein-cholesterol (LDL-C)/high-density lipoprotein-cholesterol (HDL-C) atherogenic index. The test item’s effect was beneficial on decreasing not only the LDL-C/HDL-C index but also serum total cholesterol levels in the male cohort [55].

In terms of potential antidiabetic properties of whey peptides, the concentration–time curves of glucose and insulin were monitored after a supplementation of 1.4 g of the test item over 6 weeks by prediabetic patients (n = 21, mean age of 62 years old). Authors reported a significant impact on a postprandial blood glucose profile with more glycemic than insulinotropic properties [56]. Similarly, Saleh et al. [57] aimed to demonstrate the antidiabetic effect of casein (17 g/d, over 8 days, mean age of 31 years old) in patients with mild gestational diabetes (n = 26). Among the parameters assessed (plasma glucose, insulin and C-peptide levels, and the insulin-to-glucose ratio), no insulinotropic effects occurred, but a moderated reduction of plasma glucose levels occurred.

However, Jensen et al. [37] showed that, in a randomized, double-blind study with adults suffering from metabolic syndrome (n = 15, mean age of 53 years old), the supplementation with 4 g of cod peptides over eight weeks had an impact on the levels of high-sensitivity C-reactive protein, whereas the other parameters evaluated (e.g., fasting and postprandial levels of acylated ghrelin, and fasting levels of adiponectin, leptin) did not show any statistically significant differences. These authors also reported that two months supplementation with 4 g of cod protein hydrolysate had no effect on fasting or postprandial levels of insulin, glucose or GLP-1, lipid profile, or body composition in individuals (n = 15) suffering from metabolic syndrome [58].

Tackling the risk factors from the beginning is crucial in order to reduce the risk of inflammation, especially upon the ingestion of different food components. Protein has shown to have a beneficial effect, as the peptides can exert their action in different targets. However, the mechanisms of action of the interactions occurring are not fully understood, and studies with enhanced methodology and more clear results are required.

### 3.6. Effect on Blood Pressure

The relationship between hypertension and proinflammatory cytokines, as well as the cells of the innate and adaptive immune systems, has been shown. In this regard, the sympathetic nervous system, which is described as a key determinant of hypertension, stimulates the bone marrow, spleen, and peripheral lymphatic system, and is proinflammatory. Additionally, an increase in the cytokine levels leads to structural changes in the resistance vessels, causing elevated blood pressure [59].

Concerning the antihypertensive potential of peptides, Musa-Veloso et al. [60] carried out a randomized, double-blind, placebo-controlled, multicenter trial to evaluate whether 1.2 g of shrimp peptides could have an effect on adults with mild to moderate hypertension (n = 144, mean age of 55 years old). Authors indicated a significant reduction of blood pressure, possibly due to a reduction in angiotensin II levels.

The intake of 3 g of peptides obtained from egg ovalbumin over 12 weeks (including an intermediate wash-out period) from middle-aged people (n = 70, mean age of 57 years old) with high to normal blood pressure was evaluated for the antihypertensive effect, considering the evaluation of blood pressure, arterial stiffness anthropometry, plasma lipids, and glucose. However, the results obtained did not lead to any decrease in blood pressure, nor any improvement in markers of cardiovascular diseases risk [61]. On the other hand, the use of plant-derived peptides has been growing in recent years but only a study with rice bran peptides (43 µg of the peptide LRA ingested daily for 12 weeks) was reported, aiming to determine hypotensive effects in middle-aged people with high to normal blood pressure or grade 1 hypertension (n = 50, mean age of 54 years old). According to the authors, a decrease of systolic blood pressure was observed, while no serious adverse events occurred [62].

Hypertension is currently one of the most problematic diseases in modern societies, as it is directly correlated with the development of cardiovascular disease. The efficacy of peptides to regulate blood pressure in humans might be one promising non-medical strategy to reduce the worsening of risk factors. Inflammation markers could add value to the explanation of the changes in blood-pressure-related parameters, in order to understand its complete relationship with the immune system.

### 3.7. Effect on Neurological Parameters

Neurological disorders (e.g., epilepsy, dementia, motor neuron diseases, headache disorders, sleep disorders) related to inflammation are a growing problem in modern societies. The pathogenesis of these diseases is related to a dysregulation of the immune system, and can cause immune activation of the central nervous system (CNS), leading to an increase of cytokine levels and/or infiltration of immune cells into the CNS [63]. An adequate management of the inflammation is subsequently crucial in order to have a healthy ageing and avoid the development of neurological problems. Two studies related to memory in humans were identified, although the parameters evaluated did not take into account inflammatory markers.

A chicken extract (670 mg) was orally administered to healthy middle-aged people under mild stress (n = 90, mean age of 42 years old) for 8 weeks in order to assess changes in neurocognitive tests and biochemistry markers. An improvement in verbal short- and long-term memory and spatial working memory was found [64]. In another study performed in healthy adults (n = 76, mean age of 55 years old), the effect of the intake of silk fibroin peptides (0, 280, 400, or 600 mg) over 3 weeks was assessed. The endpoints considered were a learning and memory test, learning gradient, memory maintenance, retrieval efficacy, and drawing/recall scores. A dose-dependent amelioration of the memory quotient score, the learning gradient, the numbers of words remembered, the retrieval efficiency, and drawing/recall was recorded, while no adverse effects were reported [65].

Evaluating whether a diet has an impact on the development of neurological diseases is a challenge, and the identification and validation of markers appearing at early stages could be one approach to enhance the relevance of the studies in order for them to be applied and evaluated properly.

## 4. Challenges of Peptides as Immune-Promoting Agents

As reviewed before, there are many clinical trials aiming to demonstrate the health benefits of ingesting protein hydrolysates in human physiology. However, there are still many challenges to be addressed towards which research should go, especially in how these bioactive peptides might impact the immune system of individuals (healthy or not). There are other potentially relevant studies in demonstrating the effectiveness of these protein hydrolysates, but will not be discussed in-depth since they consist of acute ingestion of the test item [66,67] or the supplementation being in combination with other compounds considered bioactive [68,69].

The first and more evident problem in the approval of peptides as health-promoting ingredients is the lack of studies carried out in humans that are well-designed and clearly demonstrating a dose–response relationship of specific endpoints, exclusively based on the intake of peptides. In line with that, multi-center analyses are needed in order to confirm the effects with scientific solidity, as well as a meta-analysis summarizing the most relevant changes. Studies carried out in vitro or on animals (e.g., in mice, rats, rabbit) are useful but they can only provide results on the mechanisms underlying, not in proving health benefits, as the whole physiology of human beings are not considered.

However, from this major challenge, several points have to be considered: (i) whether the dose ingested needs to observe a health benefit that makes it plausible for human consumption in the long term, mostly considering safety; (ii) peptides have been associated with bitter taste, due to their hydrophobic character [70], though this is something usually not addressed in human studies, or it is masked with flavorings; (iii) at the moment, scarce data are available on the interactions that bioactive peptides might have with drugs or other food components in the organisms; (iv) the evidence on the mechanisms of action of peptides is insufficient and considering that most human studies are done with protein hydrolysates, the specific function of each peptide is hard to describe and define; (v) in line with this, the toxicokinetic properties (absorption, distribution, metabolism, and excretion) should be clearly stated for bioactive peptides, as sometimes physiological changes are observed but the bioavailability of these compounds cannot be demonstrated.

On top of that, in the production process of bioactive peptides, there are also some challenges such as a consistent batch-to-batch variation in order to have a high-quality and consistent product at an industrial level, or the absence of stability data during storage, as well as the potential problems that might be encountered when entering the market, due to the unfamiliarity of consumers with the term “hydrolysate.”

The duration of the studies is not always in compliance with the guidelines, and consequently, the relevance of the results of these studies can only be used as supporting evidence, but never as solid scientific evidence of a health benefit. One challenge that has to be addressed by the scientists is the methodology employed in the studies, considering the treatment of samples and the limits of detection/quantification of some assays, as well as the accuracy of the results.

The complexity of the immune system and its relations with the body at cell, tissue, and organ levels in several pathways [71,72] hinders the ability to draw global conclusions on how the intake of bioactive peptides has a positive impact on the whole organism. The precise mechanism of immunomodulation of peptides is not entirely described yet. However, it has been described that these compounds would act mostly through triggering macrophages, the stimulation of phagocytosis, an increase in leukocyte counts, enhanced induction of immune modulators NK cells stimulation, stimulatory effect on splenocytes, CD4+, CD8+, CD11b+, and CD56+ cells, activation of transcription factor nuclear factor-κB (NF-κB), and mitogen-activated protein kinase (MAPK)-dependent pathways and the inhibition of pro-inflammatory mediators [9].

Regarding new strategies these peptides could serve, a recent study conducted on the effect of bioactive peptides on COVID-19 showed that peptides can mitigate type II transmembrane serine proteases inhibition, furin cleavage, and renin-angiotensin-aldosterone system members, concluding that bioactive peptides might have potential activity against SARS-CoV-2 infection [73,74,75].

There are protein hydrolysates from sources that have been evaluated at in vitro levels for their immunomodulatory properties, but no clinical trials were found using them as test items, such as insects [76] or mushrooms [77], although literature showed that their derived proteins could also contain bioactive peptides potentially exerting immunomodulatory effects. It is expected that clinical trials employing alternative proteins will be carried out soon and will pave the way to employ these protein sources as ingredients in the food industry [78]. The multi-functionality of peptides and how these, if bioavailable, can have a relevant impact on human physiology is still to be unraveled, and potentially new targets will be identified in the near future. The clinical trials should be properly designed, following guidelines, in order to clearly state the benefits [79].

Finally, the methodology employed in the analysis of the samples might be different among studies, creating a bias in the comparison [80]. Standardized methodologies in order to evaluate biologically relevant parameters, with validated methods, is also a challenge to be addressed by scientists in order to improve the reliability of the results [81].

## 5. Conclusions

Human studies are needed in order to demonstrate health benefits of bioactive peptides. Even when in vitro, cell-based and animal studies have shown that these products can modulate several physiological processes and potentially impact the immune status of subjects. In this review, a recent summary of studies indicating the most relevant parameters (dose, source, time, endpoints measured, and outcomes) have been described. In addition to that, the potential limitations of some of these studies have been highlighted. On top of that, it is on the weight of evidence, considering experts’ judgement, that immunomodulatory properties must be assessed. This review does not aim to indicate whether any of the studies mentioned can be considered as sufficient or inconclusive evidence to support health claims.

The effect of food-derived peptides on the health status of individuals can occur at different levels, targeting several endpoints, depending on the sequences. A proper identification of the peptides exerting specific activities has not been described, considering the technical limitations of the methodology currently available. Nonetheless, changes in relevant parameters have indicated that peptides from many sources can have an impact on some subjects. However, more research related to mechanisms of action and more controlled studies are still required in order to state that a protein hydrolysate might have immunomodulatory properties in humans.

## Data Availability

No new data were created or analyzed in this study. Data sharing is not applicable to this article.
